# Mechanisms of Proteinuria in HIV

**DOI:** 10.3389/fmed.2021.749061

**Published:** 2021-10-13

**Authors:** Gentzon Hall, Christina M. Wyatt

**Affiliations:** ^1^Department of Medicine, Division of Nephrology, Duke University School of Medicine, Durham, NC, United States; ^2^Duke Molecular Physiology Institute, Durham, NC, United States; ^3^Duke Clinical Research Institute, Durham, NC, United States

**Keywords:** podocyte, glomerular disease, HIVAN-associated nephropathy, APOL1, collapsing FSGS

## Abstract

Proteinuria is common in the setting of HIV infection, and may reflect comorbid kidney disease, treatment-related nephrotoxicity, and HIV-related glomerular diseases. The mechanisms of podocyte and tubulointerstial injury in HIV-associated nephropathy (HIVAN) have been the subject of intense investigation over the past four decades. The pathologic contributions of viral gene expression, dysregulated innate immune signaling, and ancestry-driven genetic risk modifiers have been explored in sophisticated cellular and whole animal models of disease. These studies provide evidence that injury-induced podocyte dedifferentiation, hyperplasia, cytoskeletal dysregulation, and apoptosis may cause the loss of glomerular filtration barrier integrity and slit diaphragm performance that facilitates proteinuria and tuft collapse in HIVAN. Although the incidence of HIVAN has declined with the introduction of antiretroviral therapy, the collapsing FSGS lesion has been observed in the context of other viral infections and chronic autoimmune disorders, and with the use of interferon-based therapies in genetically susceptible populations. This highlights the fact that the lesion is not specific to HIVAN and that the role of the immune system in aggravating podocyte injury warrants further exploration. This review will summarize our progress in characterizing the molecular mechanisms of podocyte dysfunction in HIVAN and other forms of HIV-associated kidney disease.

## Introduction

In the four decades since the first cases of AIDS were reported in 1981, an estimated 77.5 million people have been infected with HIV and more than 34 million people have died from complications of HIV infection (1). Kidney disease emerged as an important complication of HIV in the early years of the epidemic, with the first reports of a unique pattern of collapsing focal segmental glomerulosclerosis (FSGS) with accompanying tubulointerstitial injury published in 1984 (2, 3). HIIV-associated nephropathy (HIVAN) quickly became the leading cause of end-stage kidney disease (ESKD) in people living with HIV (PLWH), demonstrating a marked predilection for individuals of African descent. Although the incidence of ESKD attributed to HIVAN plateaued in the United States following the widespread introduction of 3-drug antiretroviral therapy (ART) in 1997, HIVAN remains an important cause of kidney disease in the setting of untreated HIV infection (4). The original case series also reported a spectrum of immune complex glomerular lesions, and contemporary biopsy series continue to identify immune complex kidney diseases as one of the most common histologic diagnoses in PLWH. Other common causes of kidney disease in PLWH include ART toxicity and comorbid kidney disease due to traditional risk factors such as diabetes (4). As a result, kidney biopsy is often required for definitive diagnosis of proteinuric kidney disease in PLWH.

The epidemiology of HIVAN suggested that both viral and host factors play a central role in pathogenesis. The development of HIVAN in HIV-transgenic mouse models has allowed for extensive investigation into the mechanisms of glomerular injury, proteinuria, and kidney failure in HIVAN, which will be the primary focus of this review.

## HIV Infection of the Kidney

**Figure 1:**
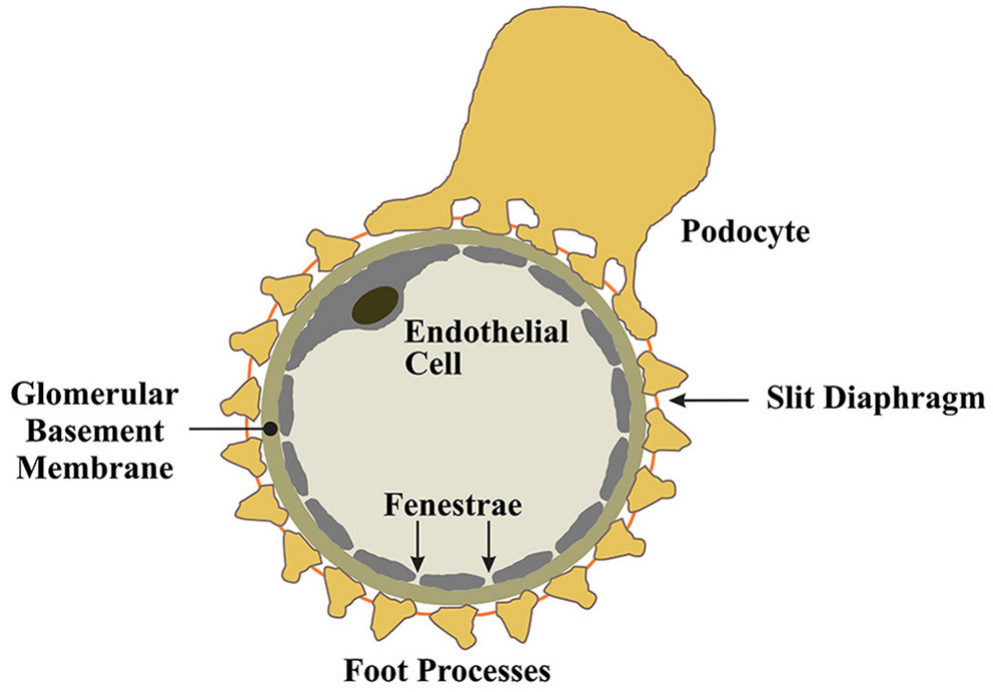
Mechanisms of proteinuria in HIVAN.

**Figure 2:**
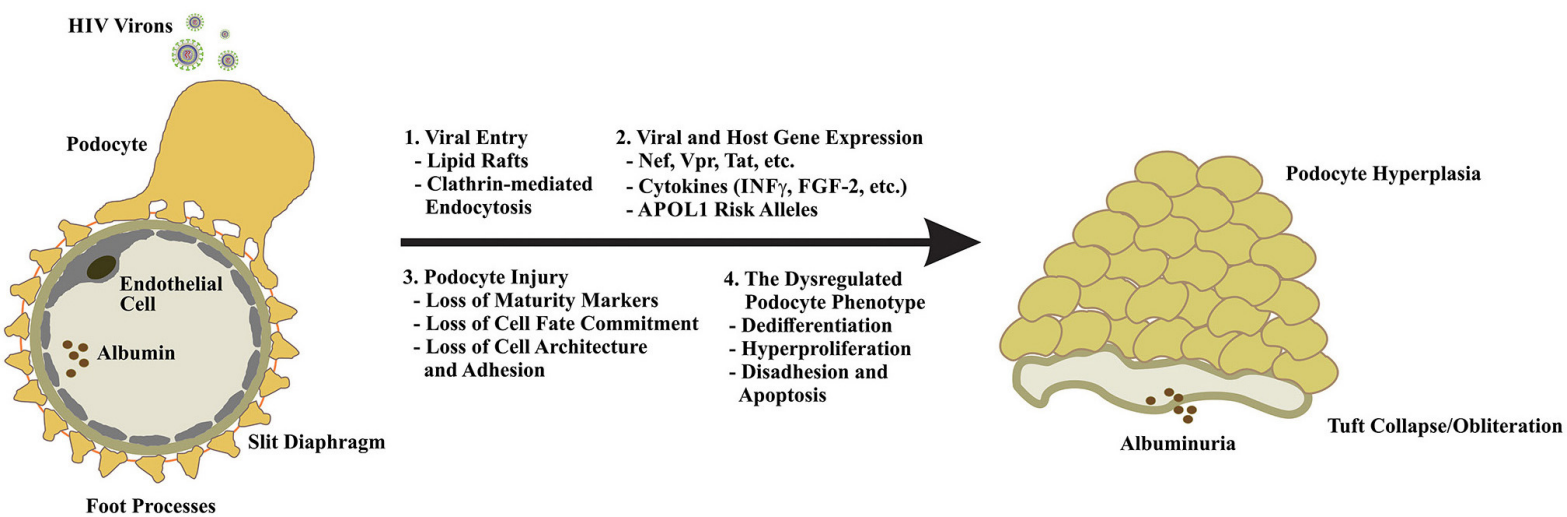
Podocyte dysfunction in response to HIV infection is multifaceted. Because podocytes lack the receptors for canonical viral entry, viral particle entry via lipid rafts, and by clathrin-mediated endocytosis have been proposed (1). Upon entry, expression of viral genes and other mediators of innate immunity drive podocyte cytotoxicity and may enhance the expression of the high-risk APOL1 alleles in individuals of African descent to increase their risk of developing HIVAN (2). Podocyte injury manifests in the loss of maturity markers (e.g., nephrin, synaptopodin, WT1, CALLA, etc.), loss of cell fate commitment, and hyperplasia and the loss of cytoarchitecture and adhesion (3). The dysregulated podocyte phenotype appears to be irreversible and largely unresponsive to standard-of-care FSGS therapies highlighting the urgent need for a nuanced understanding of disease evolution, novel therapeutics, and early intervention (4).

The emergence of HIVAN in the setting of AIDS and the decline in incidence of ESKD with the introduction of ART is consistent with a direct role for HIV in the pathogenesis of HIVAN. Early reports demonstrated the presence of HIV nucleic acids in renal epithelial cells (5–7); however, the absence of CD4, CXCR4, and CCR5 receptor expression on these cells (8, 9) implied the existence of a non-receptor mediated viral entry mechanism (10, 11). The subsequent identification of HIV-1 entry into human podocytes via lipid rafts (12) and by dynamin-mediated endocytosis (13–15) provided compelling evidence for non-canonical routes of viral particle entry. Although many questions remain, substantial progress has been made in characterizing the effects of HIV infection on podocyte physiology and function (Figures 1, 2).

## Normal Podocyte Function at the Glomerular Filtration Barrier

Podocytes are an essential cellular component of the tripartite glomerular filtration barrier (16). Podocytes are post-mitotic epithelial cells characterized by their highly specialized, actin-based membranous extentions known as foot processes (16). Podocytes envelop glomerular capillaries, adhering to the glomerular basement membrane (GBM) through a network of intermolecular interactions connecting foot processes to the GBM (16). Between interdigitating foot processes, a zipper-like assembly of proteins known as the slit-diaphragm functions as a molecular seive to provide charge and size selectivity for ultrafiltration (17–19). Podocytes produce the molecular constituents of the slit diaphragm and the expression of these proteins coincides with podocyte differentiation and maturation (20, 21). For example, the slit diaphragm protein nephrin, first identified in a Finnish cohort study of congenital nephrotic syndrome (17, 22), is produced by podocytes. The various roles of nephrin at the slit diaphram and as a modulator of prosurvival signaling in podocytes are well-documented (23–28) and will not be detailed in this review, however, it is clear that disease processes that impair podocyte nephrin expression, and other slit diaphragm components, result in podocyte dysfunction and drive the development of proteinuria and the FSGS lesion (29).

## Effects of Viral Gene Expression on Podocyte Phyisology and Function

The collapsing FSGS lesion of HIVAN is characterized by podocyte dedifferentiation and hyperplasia, loss of podocyte maturity markers, foot process effacement and podocyte detachment, podocyte apoptosis, and heavy proteinuria (30–33). The cytotoxic effects of HIV gene expression in podocytes are well-established, and experimental models of HIVAN suggest that podocyte-restricted expression of viral proteins is sufficient to induce a dysegulated podocyte phenotype and the collapsing FSGS lesion (30, 31, 34–38). In particular, subtantial evidence exists for the roles of the HIV proteins Nef and Vpr in driving podocyte injury and dysfunction in HIVAN (Figures 1, 2).

### Nef

Nef is one of four accesory proteins (i.e., Nef, Vpr, Vif, and Vpu) expressed by HIV (39). Despite early descriptions of Nef as a negative regulatory factor of viral replication (40–42), subsequent studies demonstrated that Nef exerts a neutral or positive effect on viral replication in various cell types (39, 42). Although HIV does not appear to produce productive infection in podocytes (13, 43), Nef exerts a variety of deleterious effects on podocyte physiology and function that are unrelated to the enhancement of viral replication. In 2002, Husain et al. demonstrated that Nef expression induced the loss of maturity markers, proliferation and anchorage-independent growth in cultured human podocytes (36). These data were later validated in a murine model of podocyte-restricted Nef expression. Husain et al. showed that podocyte-specific expression of Nef caused the loss of maturity marker expression (i.e., synaptopodin and WT1), induction of STAT3 activtion, and expression of the proliferation marker Ki-67 (36). Notably, this model did not manifest the proteinuria or glomerular injury chracteristic of HIVAN, leading the authors to conclude that Nef may be responsible for the early molecular changes that drive podocyte injury in HIVAN. Sunamoto et al. demonstrated that Nef expression was necessary and sufficient to induce proliferation and dedifferentiation in murine podocytes (44). He et al. later provided mechanistic insights into the role of Nef in podocyte hyperplasia when they demonstrated that Nef stimulates pro-proliferative signaling through the Src tyrosine kinase-dependent activation of Ras-c-Raf-MAPK1/2 and STAT3 signaling in conditionally immortalized human poocytes (45). The importance of STAT3 activation in podocyte hyperplasia was highlighted by the work of Feng et al. who demonstrated that reduction of STAT3 expression and activity ameliorated proteinuria, glomerulosclerosis, and tubulointersitial injury in a murine model of HIVAN (46). Similar findings were also reported by Gu et al. with STAT3 gene deletion in the same animal model (47). STAT3 is an established transcriptional regulator of molecules that drive cell-cycle re-entry and proliferation such as C-Myc, Cyclin D-1, CDC25A, and anillin (48, 49), supporting the hypothesis that STAT3 is a key regulator of podocyte proliferation in HIVAN. Several studies have also implicated Nef in the disruption of the podocyte cytoskeleton through various intermolecular interactions with actin and other key regulators of cytoskeletal dynamics (50–55). Other functions of Nef, such as its ability to interact with clathrin at the plasma membrane to disrupt endocytic trafficking, may also contribute to podocyte injury; however, this aspect of Nef signaling has not been documented in podocytes.

### Vpr

The HIV accessory protein Viral Protein R (Vpr) has also been identified as a significant contributor to kidney injury in HIVAN. Like Nef, podocyte-restricted expression of Vpr in murine models established on the susceptible FVB/N background was sufficient to produce glomerular collapse and tubulointerstitial disease (38, 56, 57). Double transgenic expression of Vpr and Nef synergistically induced the full spectrum of podocyte injury, glomerular collapse, and tubulointerstitial diseased observed in human HIVAN (38, 57). In renal tubular epithelial cells (RTECs), Vpr has been shown to induce G2/M phase cell cycle arrest and dysregulation of cytokinesis (57–60). Vpr also induces apotosis in RTECs via the persistant activation of ERK MAP kinase and the upregulation of the ubiquitin-like protein FAT10 (61, 62). Less is known about the mechanisms of Vpr-induced podocyte injury. In 2014, Gbadegesin et al. demonstrated that the cytokinesis regulatory protein and pro-proliferative signaling molecule anillin, was upregulated in a murine model of podocyte-restricted Vpr expression (63). Anillin is an essential component of the cytokinetic ring and a driver of abnormal cellular proliferation in various malignacies (64–66). In the Vpr transgenic mouse, the upregulation of anillin in glomerular podocytes likely represents an accumulation of anillin in arrested cells or a cell-type specific derangement of cytokinetic drive and cell-cycle re-entry signaling.

### Other Viral Proteins

The HIV regulatory protein Tat may also contribute to podocyte dysfunction in HIVAN. Tat is essential for HIV gene transactivation (67). In primary and conditionally immortalized podocytes, Conaldi et al. showed that Tat expression induced basic fibroblast growth factor (FGF-2)-driven hyperplasia, loss of maturity markers, cytoskeletal dysregulation, and impairment of permselectivity in a dose-dependent manner (43). Similar findings were later reported by Doublier et al. who showed that Tat exposure impaired the permeability of isolated glomeruli and reduced nephrin expression in conditionally immortalized human podocytes (68). Insights into the mechanisms of Tat-induced podocyte injury were provided by Xie et al., who reported that Tat targets to cholesterol-enriched lipid rafts, where it drives RhoA, matrix metalloproteinase-9 expression and FGF-2-mediated proproliferative signaling (69). Notably, murine models of podocyte-restricted Tat expression have failed to recapitulate the HIVAN phenotype (70). Overexpression of other HIV proteins such as Rev, Vif, and Vpu have not been associated with podocyte cytotoxicity and have not induced glomerular injury in murine models (70).

### Contributions of the High-Risk Apolipoprotein L1 (APOL1) Alleles

The epidemiology of HIVAN is also consistent with a role for host genetic susceptibility, with a marked predilection for individuals of African descent. The discovery of high-risk variants in the *APOL1* gene provided evidence of a genetic contribution to the racial disparity (71). The G1 (rs4821481 and rs3752462) and G2 (rs71785313) *APOL1* variants were identified in an association analysis comparing 205 African-American individuals with non-familial, biopsy-proven FSGS and 180 healthy African-American controls. *APOL1* encodes apolipoprotein L1, a trypanolytic serum factor that confers resistance against the parasitic infection that causes African sleeping sickness (71). The G1 and G2 *APOL1* variants are found exclusively in individuals of recent African descent and confer resistance against a deadly subspecies of Trypanosoma that is normally resistant to lysis by wild-type APOL1. Carrying two *APOL1* variants significantly enhances the risk of developing HIVAN in untreated HIV-infected individuals and explains up to 35% of the disease (72, 73). Our understanding of the mechanisms of APOL1-mediated kidney injury is rapidly increasing. In 2016, Olabisi et al. identified direct cytotoxic effects of the APOL1 proteins via the formation of cation permeable pores that disrupt potassium flux and lead to cellular swelling and death (74). Subsequently, Jha et al showed that the APOL1-mediated enhancement of potassium efflux induces proinflammatory cytokine expression, activation of the NLRP3 inflammasome and cellular pyroptosis (75). Other mechanisms of APOL1-mediated cellular injury have been uncovered. For example, Ma et al demonstrated that the APOL1 renal risk variants induce mitochondrial fission, reduce mitochondrial repiratory capacity, respiration rate and membrane potential (76, 77). Expression of the G1 and G2 variants also induced dysregulation of endosomal trafficking and lysosomal acidification in Drosophila and Saccharomyces (78). Additionally, the G1 and G2 renal risk variants have been shown to enhance the expression of miR193a, a negative regulator of autophagy (79). Consistent with an impairment in autophagy, Wen et al showed that overexpression of the APOL1 risk alleles induce endoplasmic reticulum stress in culture human podocytes (80). Upregulation of miR193a has also been shown to impair adherens complex stability, disrupt actomyosin cytoskeletal organization, reduce nephrin expression and promote dedifferentiation in podocytes (79, 81).

In the context of HIVAN, elaboration of interferon-γ (INF-γ) and other circulating mediators of innate immunity signaling appear to drive *APOL1* gene transcription (82, 83). *In vitro*, the cytotoxicity of the high-risk *APOL1* variants is dose-dependent, suggesting that any process that enhances the expression of the *APOL1* renal risk alleles may provoke glomerular injury (84). This finding may, at least partially, explain why HIV is among the strongest promoters of glomerular disease in the setting of the high-risk *APOL1* genotype, which has been associated with up to 89-fold increase in odds of HIVAN (85). Notably, collapsing glomerulopathy has been observed in individuals of African descent treated with interferon therapies and following viral infection with Parvovirus B19, CMV, EBV, HTLV1, Coxsackie B, Dengue, Zika, and most recently, SARS-CoV-2 (86–88). Small studies have demonstrated an association between the high-risk *APOL1* genotype and the development of collapsing FSGS in the setting of COVID-19 (89) (Figures 1, 2).

## Mechanisms of Proteinuria in Other Kidney Diseases in PLWH

HIV infection, by a variety of intracellular and systemic influences on podocyte physiology, perturbs cellular fate commitment, gene expression, and viability to promote development of the collapsing FSGS lesion of HIVAN. A nuanced understanding of the processes that drive podocyte injury in HIVAN may uncover novel therapeutic targets for treatment of other glomerular diseases. Prompted by epidemiologic studies demonstrating accelerated progression of kidney disease in the setting of HIV and diabetes (90), Mallipattu et al. demonstrated that the induction of diabetes with streptozotocin resulted in more prominent histologic changes in HIV-transgenic mice compared to wild-type littermates (91). These findings were confirmed in a subsequent study using podocyte-specific transgenic mice with low HIV transgene expression to more closely reflect the current clinical status of ART-treated individuals (92). In this model, HIV and diabetes had a synergistic effect on the expression of Sirtuin-1 deacetylase, suggesting a potential therapeutic role for Sirtuin-1 agonists.

A lack of animal models has slowed progress toward elucidating the pathogenesis of immune complex glomerular disease in PLWH. This has been compounded by the diverse spectrum of glomerular lesions that occur in this setting and that have been considered together in most clinical studies. Small but rigorous human studies have suggested a role for immune complexes directed against HIV antigens in the pathogenesis of immune complex kidney disease (93). Because podocytes have been shown to play a role in the clearance of immune deposits (94), it is possible that HIV-induced podoycte damage also promotes immune complex kidney disease in PLWH.

Kidney injury due to the antiretroviral agent tenofovir disoproxil fumarate (TDF) may also present with proteinuria, although this is typically low molecular weight proteinuria rather than albuminuria. Tenofovir is a nucleotide analog that is chemically related to the older antiviral agents cidofovir and adefovir, both of which are known to exhibit dose-limiting proximal tubular toxicity. The first approved tenofovir prodrug, TDF, has been associated with proximal tubulopathy and non-albumin proteinuria. Although the mechanism of proximal tubular cell injury has not been fully elucidated, it is thought to involve mitochondrial dysfunction as a result of the weak inhibition of mitochondrial DNA polymerase gamma (95, 96). Tenofovir is eliminated by glomerular filtration and active proximal tubular cell secretion, and an increase in intracellular concentration due to increased plasma concentration, decreased glomerular filtration, or impaired apical transport of tenofovir is thought to increase the risk of proximal tubular cell dysfunction or injury. Although it is possible that HIV-induced cell damage promotes tenofovir toxicity, non-albumin proteinuria has also been observed with the use of TDF for HIV pre-exposure prophylaxis in HIV-negative individuals (97). A newer prodrug, tenofovir alafenamide, is effective at lower plasma concentrations and may reduce the risk of tenofovir toxicity, although longer followup is needed.

Despite some risk of nephrotoxicity with tenofovir and other antiretroviral agents, the use of ART for treatment and prevention of HIV infection is currently the most effective way to mitigate the myriad pathogenic effects of HIV on the kidneys. While the incidence of advanced kidney disease due to HIVAN has decreased with the use of ART, HIVAN remains a valuable model for the study of podocyte injury and APOL1-induced glomerular disease.

## Author Contributions

All authors listed have made a substantial, direct and intellectual contribution to the work, and approved it for publication.

## Funding

GH was supported by the NIH/NIDDK (K08DK111940), the American Society of Nephrology and the Harold Amos Medical Faculty Development Program, the Northwestern University George M. O'Brien Kidney Research Award, the Doris Duke Charitable Foundation, and the Duke Claude D. Pepper Older Americans Independence Center. CW was supported by the NIH/NIDDK (R01DK112258 and P01DK056492).

## Conflict of Interest

GH is also a consultant for Reata Pharmaceuticals, Travere Pharmaceuticals, Otsuka Pharmaceuticals and Goldfinch Bio. The remaining author declares that the research was conducted in the absence of any commercial or financial relationships that could be construed as a potential conflict of interest.

## Publisher's Note

All claims expressed in this article are solely those of the authors and do not necessarily represent those of their affiliated organizations, or those of the publisher, the editors and the reviewers. Any product that may be evaluated in this article, or claim that may be made by its manufacturer, is not guaranteed or endorsed by the publisher.
